# Supplementary material for: Peptide-stimulated T cells bypass immune checkpoint inhibitor resistance and eliminate autologous microsatellite instable colorectal cancer cells
Source: NPJ Precis Oncol. 2024 Jul 29;8:163. doi: 10.1038/s41698-024-00645-3 (PMC11286882; doi:10.1038/s41698-024-00645-3)
Supplement: Supplementary file 1 — Supplemental material [file 41698_2024_645_MOESM1_ESM.pdf]

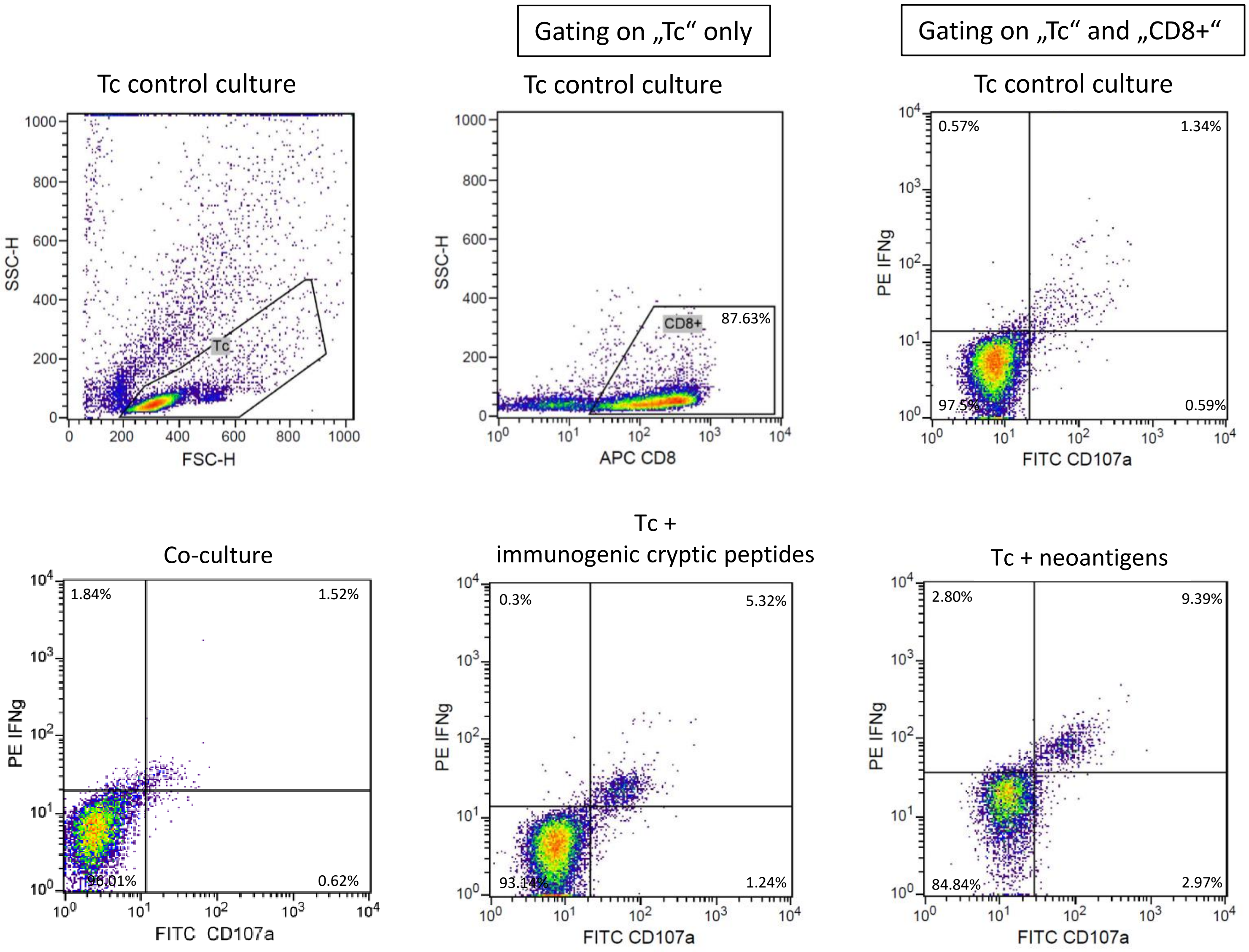

Supplementary Figure 1: Representative FACS plots of the degranulation assay. Gating strategy is shown. Depicted are measurements of pTc HROC113 stimulated for 14d with peptides, on co-culture or control culture. Gates and quadrants were defined by measurement of unstained controls.

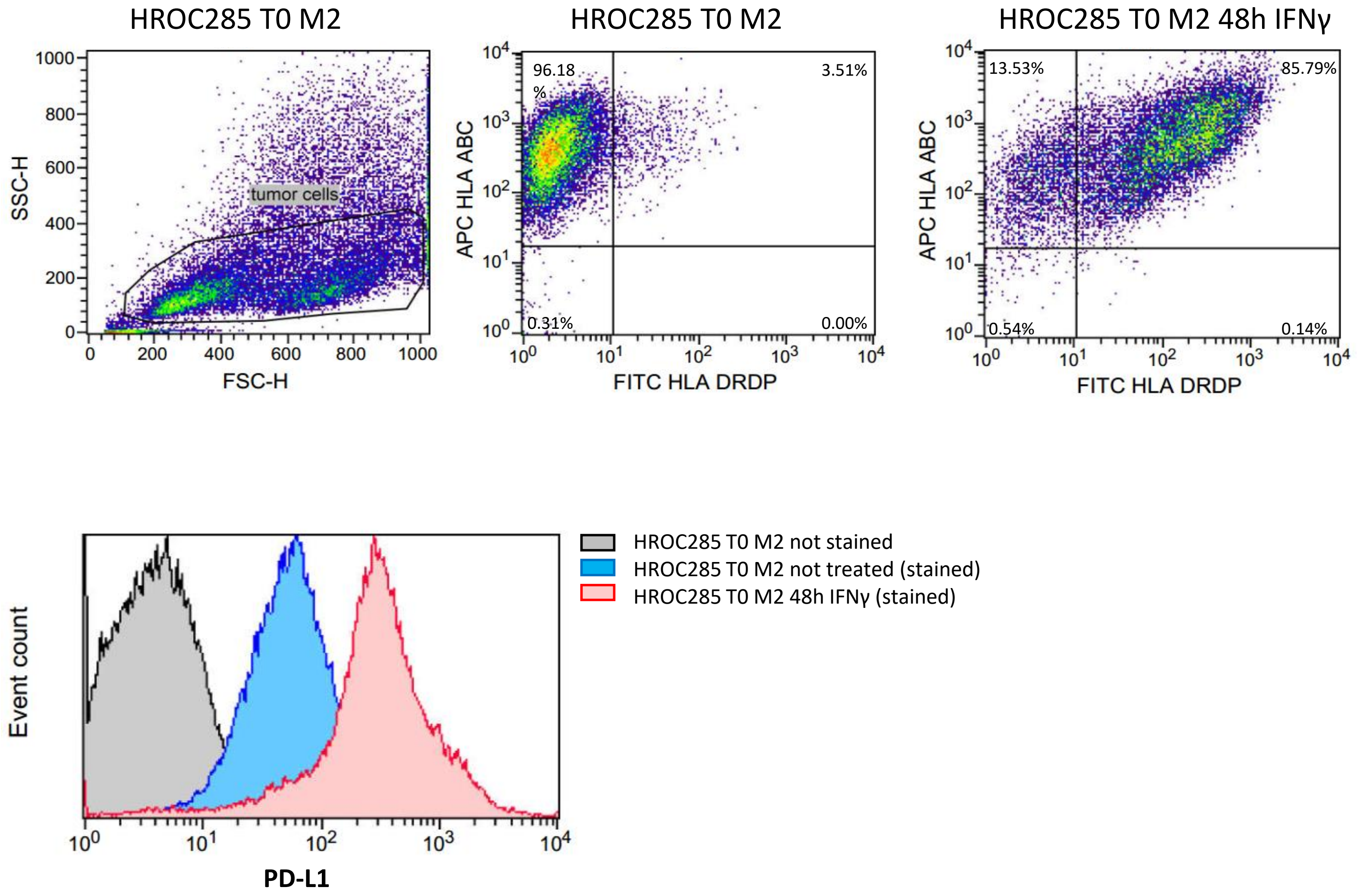

Supplementary Figure 2: Representative density plots and histograms of flow-cytometric measurements. Depicted are measurements of HROC285 T0 M2 with or without treatment of IFNγ (48h, 200IU/ml). Gates and quadrants are defined by measurement of unstained controls. (A) Densitiy plots of HROC285 T0 M2 positive for HLA ABC and HLA DRDP. (B) Median Fluorescence intensity of PD-L1 on HROC285 T0 M2.

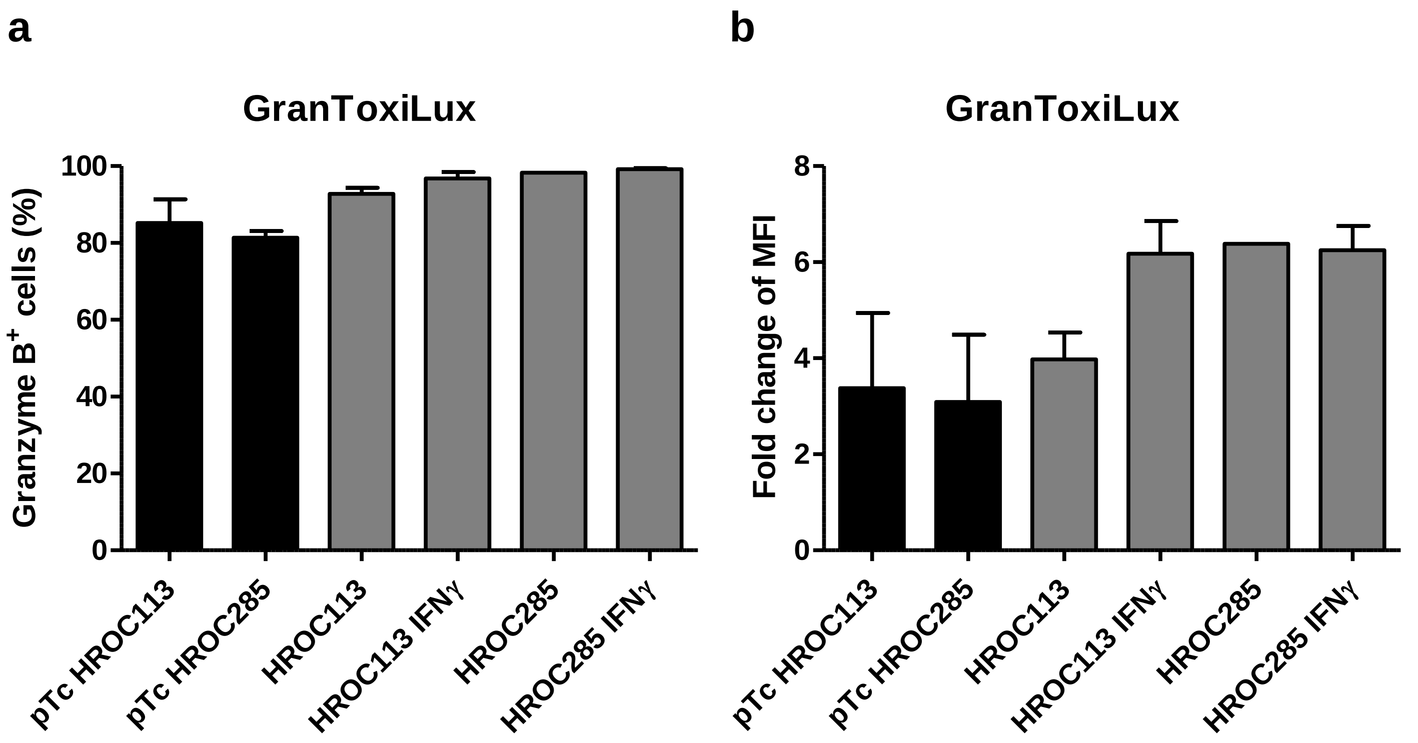

Supplementary Figure 3: Granzyme B activity in CRC cell lines. The GranToxiLux kit (Oncoimmunin Inc, Gaithersburg, Maryland, USA) was used to measure the granzyme B activity in tumor cells and pTc, which were used as positive controls. The amount of cells with granzyme B cleaving capacity was determined (A) as well as the MFI of granzyme B compared to unstained controls (B). Depicted are means of 2-3 biological replicates and the respective standard deviation.

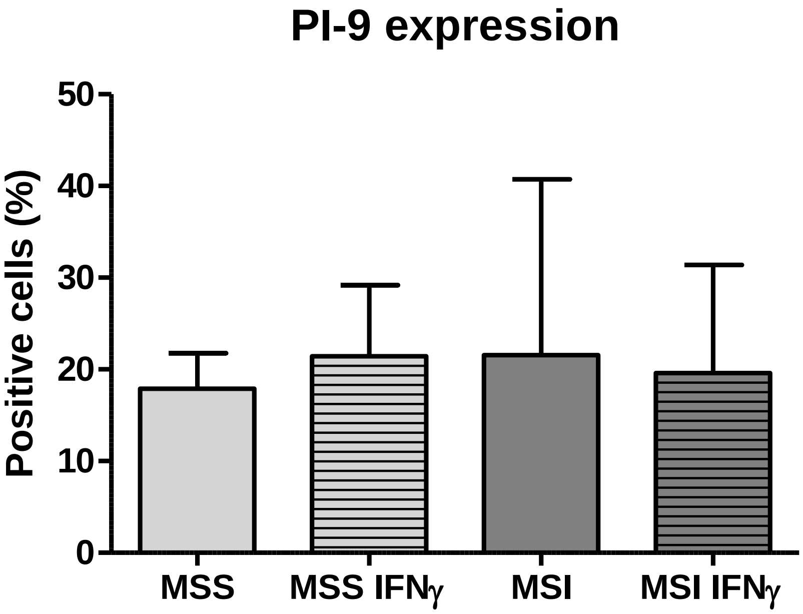

Supplementary Figure 4: PI-9 expression in microsatellite stable and unstable CRC cell lines. Comparison of percent PI-9⁺ cells between microsatellite stable (MSS; n=4) and MSI (n=5) cell lines determined by flow cytometry. Depicted are means of the biological replicates and the respective standard deviation.

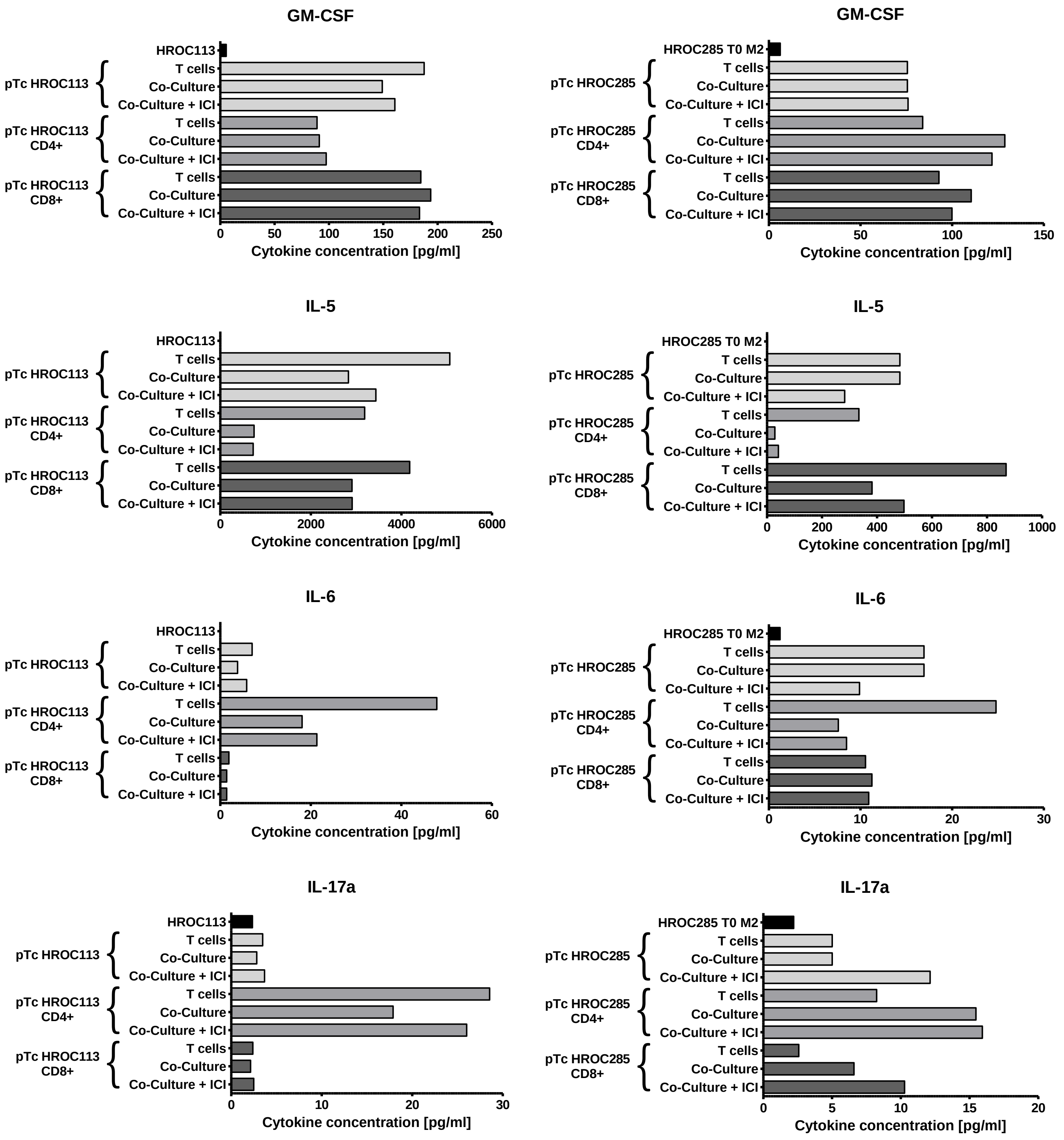

Supplementary Figure 5: Cytokine detection in cell culture supernatants of tumor cell lines. Cell culture supernatants were collected after seven days of culture or co-culture of pTc and their respective tumor cells. Cytokine concentration was determined by using the MACSPlex Cytokine 12 Kit from Miltenyi Biotec. For IL-10, IL-4, -9, -10, -12p70, IFNα and TNF, samples did not reach the limit of detection. Depicted are mean values of two technical replicates.

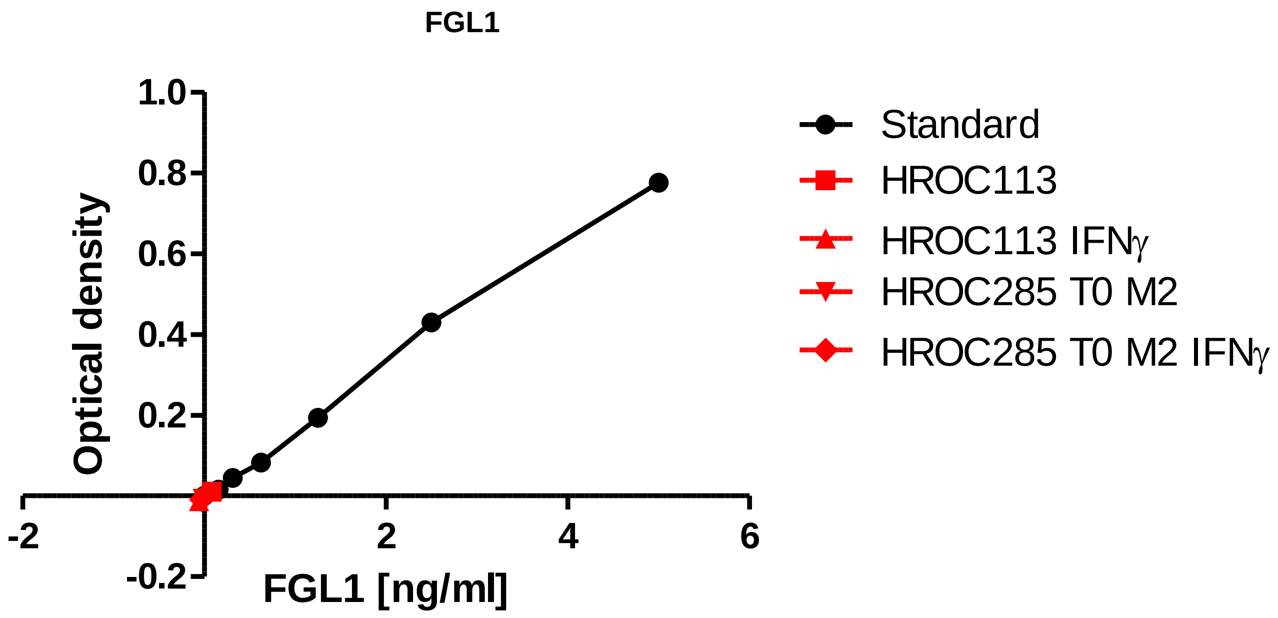

Supplementary Figure 6: FGL1 secretion by tumor cells. Tumor cells were cultivated with or without 200 IU/ml IFNγ and supernatant was collected at day four when cell growth reached confluency.

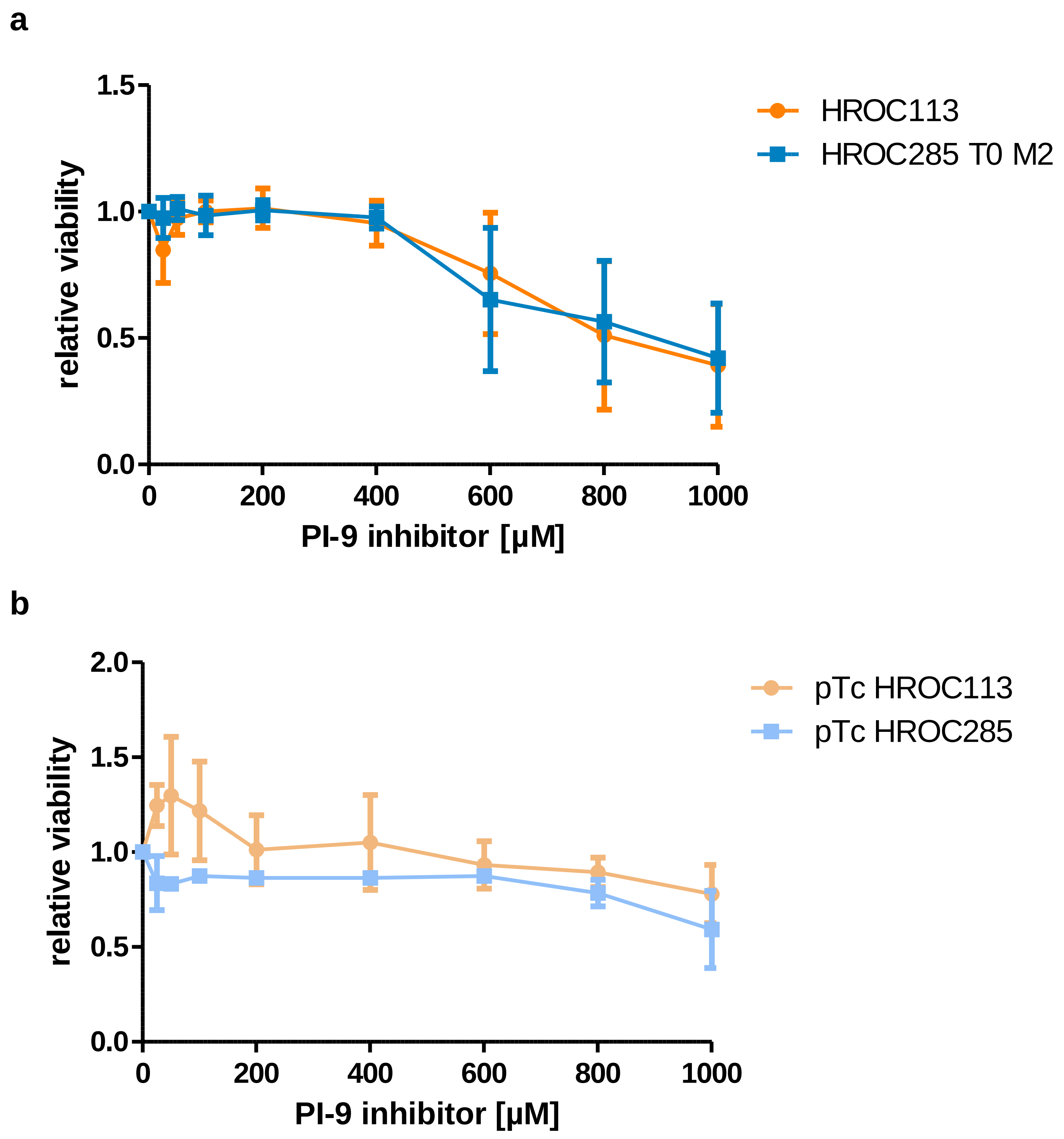

Supplementary Figure 7: Effect of PI-9 inhibitor on cells. Tumor cells (A) and pTc (B) were incubated with increasing concentrations of the PI-9 inhibitor 1,3-Benzoxazole-6-carboxylic acid for seven days. Subsequently, viability of tumor cells and T cells was determined by crystal violet staining and calcein AM assay, respectively.
